# To the Editors of the Medical and Physical Journal

**Published:** 1801-10

**Authors:** W. D. Rolfe

**Affiliations:** Dispensary House, North Street


### *To the Editors of the Medical and Phyſical Journal.*

Gentlemen,

I BEG leave to ſend you, as uſual, my month's ſtate of the patients admitted into the Briſtol Diſpenſary; and at the ſame time, to remind you of an error in my laſt paper on Typhus Fevers, wherein ℥j. is put inſtead of ʒj. of acid. muriat. in the firſt mixture, and not mentioning the ſize of the mixture, which is meant an eight ounce; by correcting the error in the next number, you will much oblige,

*Dispensary House,*

*North Street,* 10 *Sept.* 1801.

Yours, &c.

W. D. ROLFE.

